# N6-methyladenosine methylation in kidney injury

**DOI:** 10.1186/s13148-023-01586-7

**Published:** 2023-10-21

**Authors:** Qimeng Wang, Xiaoting Fan, Qinghao Sheng, Meilin Yang, Ping Zhou, Shangwei Lu, Ying Gao, Zhijuan Kong, Ning Shen, Zhimei Lv, Rong Wang

**Affiliations:** 1 Department of Nephrology, Shandong Provincial Hospital Affiliated to Shandong First Medical University, Jinan, 250021 Shandong China; 2 Department of Nephrology, Shandong Provincial Hospital, Shandong University, Jinan, 250021 Shandong China

**Keywords:** Epigenetic modification, M6A, Kidney injury, The regulation of M6A

## Abstract

Multiple mechanisms are involved in kidney damage, among which the role of epigenetic modifications in the occurrence and development of kidney diseases is constantly being revealed. However, N6-methyladenosine (M6A), a well-known post-transcriptional modification, has been regarded as the most prevalent epigenetic modifications in higher eukaryotic, which is involved in various biological processes of cells such as maintaining the stability of mRNA. The role of M6A modification in the mechanism of kidney damage has attracted widespread attention. In this review, we mainly summarize the role of M6A modification in the progression of kidney diseases from the following aspects: the regulatory pattern of N6-methyladenosine, the critical roles of N6-methyladenosine in chronic kidney disease, acute kidney injury and renal cell carcinoma, and then reveal its potential significance in the diagnosis and treatment of various kidney diseases. A better understanding of this field will be helpful for future research and clinical treatment of kidney diseases.

## Introduction

Over the past several decades, the impact of kidney disease on global health has changed dramatically. It has shifted from a subspecialty to a global health burden. Among them, the proportion of people with chronic kidney disease has far exceeded 10%, and the prevalence of high-risk groups has exceeded 50% [1]. The kidney is one of the most complex organs in the body and maintains a number of vital and important functions. Different kidney cells perform their own functions and play their own roles, but they are interconnected with each other and participate in the maintenance of kidney function. Therefore, abnormalities of different biological processes in different kidney cells are involved in the development and progression of various kidney diseases. The complexity of the kidneys also makes the diagnosis and treatment of kidney diseases difficult [2]. In order to manage kidney disease quickly and effectively and promote the development of the nephrology profession, it is essential to have a deep and extensive understanding of the biological mechanisms involved in the progression of kidney disease.

In recent years, the role of epigenetics in diseases has received extensive attention. Epigenetics refers to changes in gene expression levels based on non-genetic sequence changes. And this change is stable over the life course of the cell [3]. It includes DNA methylation, genomic imprinting, maternal effects, gene silencing, nucleolar dominance, dormant transposon activation, RNA editing and so on. Abnormalities in epigenetics not only change the epigenetic characteristics of the whole genome, but also disrupt the basic pathways that protect kidney cells from uncontrolled growth, apoptosis and the development of other kidney-related syndromes, thereby affecting the biological function of kidney cells and participating in the occurrence of kidney damage [4]. Among various epigenetic modifications, the study of M6A modifications in various diseases has recently become a hot spot. The M6A modification is a widely present post-transcriptional modification of RNA, which can be found in more than 7600 gene mRNAs and more than 300 non-coding RNAs [5]. Relevant studies have shown that M6A modifications are usually enriched around the stop codon and 3’UTR [5]. The abundance of M6A modifications is regulated by its writers, readers, and erasers, so the modification is dynamic and reversible [6]. The lack of detection methods has led to the stagnation of research on M6A modification, and in recent years, with the development of high-throughput sequencing, the research on M6A modification has gradually attracted public attention [7]. Methyl-RNA immunoprecipitation and sequencing (MeRIP-Seq, also called M6A-Seq) is a common means of studying M6A modification. This method detects the M6A peak by immunoprecipitating RNA fragments about 100-nt long with M6A-specific antibodies [8]. M6A modification works throughout almost the entire life cycle of RNA, such as alternative splicing, translation, translocation, and degradation [3]. Abnormal M6A modifications have been demonstrated to be participating in the occurrence of a variety of diseases such as cancer [9], non-alcoholic fatty liver disease [10], and inflammatory bowel diseases [11]. With the deepening of research on M6A modification, its relationship with kidney damage has been continuously revealed.

Here, we summarized the regulatory pattern of M6A modification with the participation of its writer, reader and eraser, comprehensively reviewed the role of M6A modification in renal impairment by analyzing its abnormal regulation in renal diseases and renal cell carcinoma and expounded its potential clinical significance in renal injury.

## The regulation of M6A modification

**Fig. 1:**
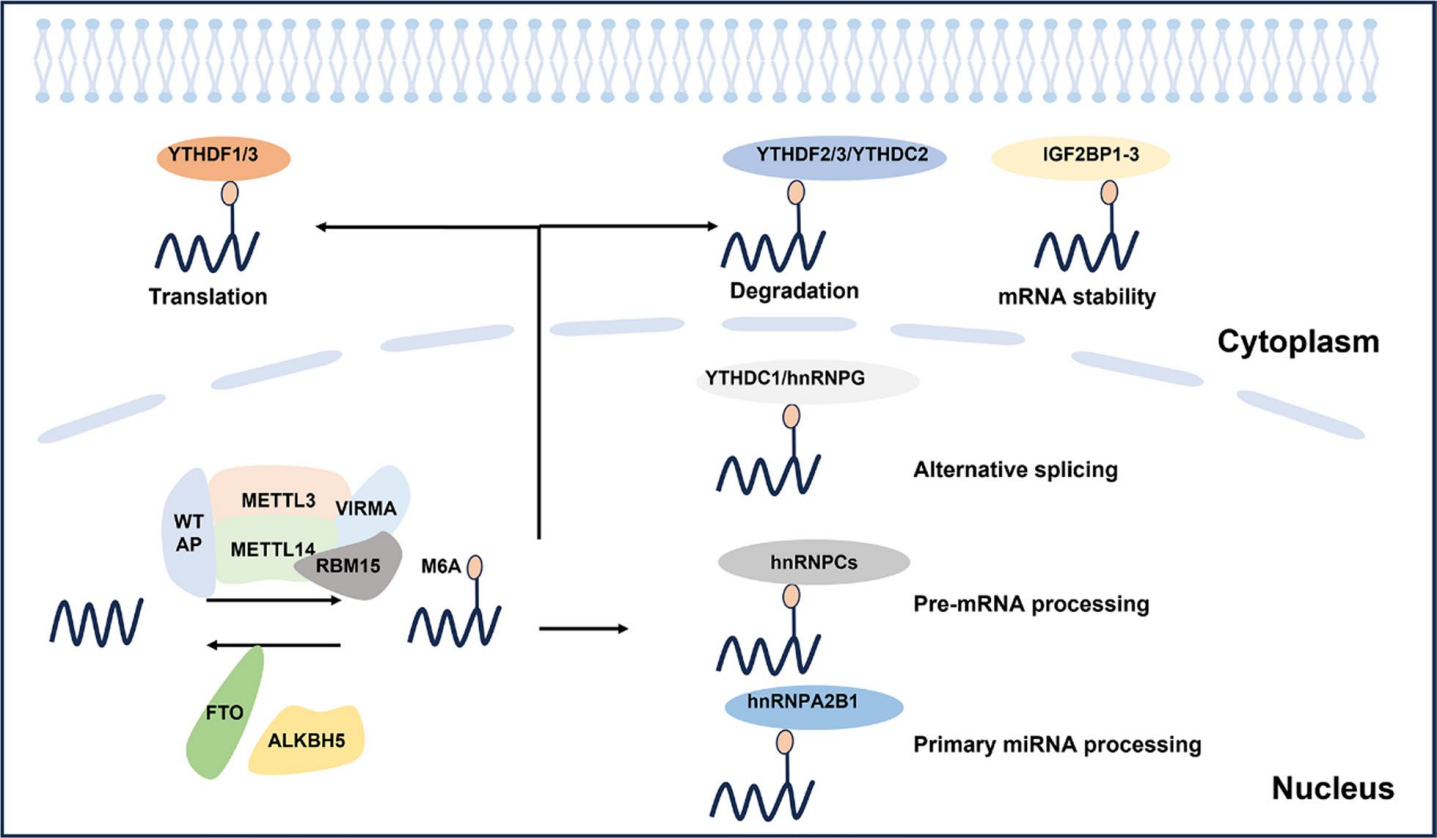
Regulators and dynamic regulation of M6A modification. RNA undergoes M6A modification by methylation transferase (METTL3, METTL14, WTAP, VIRMA and RBM15), removes M6A modification by demethylation enzymes (FTO, and ALKBH5), and performs different roles, respectively, in the presence of reading proteins (YTH family, hnRNP family and IGF2BPs)

With the regulation of its writers, readers, and erasers, the M6A modification is dynamic and reversible (Fig. 1) [6]. It is precisely these characteristics that makes the role of M6A modification in various diseases diverse and reflects its potential as the treatment target. Therefore, if we want to understand the role of M6A modification in kidney disease, we must first deeply study its molecular mechanism and regulatory mode.

### M6A writers

Methyltransferases, as M6A writers, exert M6A deposition on a variety of RNAs by their own or forming different complexes with additional partner proteins [12]. With the deepening of research, methyltransferases involved in M6A modification have been continuously discovered and confirmed. At present, the most studied methyltransferases involved in M6A modification are METTL3, METTL14 [13], WTAP [14], VIRMA and RBM15 [15]. Together, they form M6A methyltransferase complex (MTC), of which METTL3 and METTL14 are the core components [13]. The methyltransferase complex completes methylation modification by transferring a methyl group on S-adenosyl-L-methionine (SAM) to the N6 amino group of the adenosine base of RNA (M6A) [16]. The study found that the methyltransferase complex is localized to the nucleus. This mode of nuclear localization is closely related to the nuclear localization signal (NLS) on METTL3 and WTAP [17]. As a catalytic subunit, METTL3 must exert its catalytic activity in the presence of METTL14. METTL14, on the other hand, has no catalytic effect but can identify substrates [12]. The catalytic domain and CCCH-type zinc-binding motifs (ZFD) of METTL3 are critical for its promotion of target RNA methylation. Its catalytic domain has a cavity that houses the methyl donor SAM [18], while the ZFD are structures that bind to RNA substrates [19]. In addition, the N-terminal domain of METTL3 is closely related to the regulation of RNA secondary structure [20]. However, the role of METTL14 is to provide an RNA-bound scaffold for METTL3 [21]. The c-terminal RGG repeat of METTL14 is a necessary condition for the activity of the methyltransferase complex [17]. WTAP and RBM15 bind to each other to recruit MTCs into specific RNAs and achieve methylation modifications [22]. Studies have shown preferential mRNA methylation of 3' UTR and near-stop codons, and this region-selective methylation is mediated by VIRMA [23].

### M6A readers

The prerequisite for M6A-modified RNA to function is that the methyl groups it carries are recognized, and M6A readers is a class of proteins that can recognize M6A-modified RNAs. The proteins with the function of M6A-modified recognition are mainly YTH family (YT521-B homology (YTH) domain family), hnRNPs (heterogeneous nuclear ribonucleoproteins) and IGF2BPs (insulin-like growth factor 2 mRNA-binding proteins) [3]. The first M6A reader protein to be discovered was a YTH family protein [24]. The family mainly includes YTHDC1, YTHDC2, YTHDF1, YTHDF2 and YTHDF3 [25]. Three proteins in the YTHDF family exhibit different roles. The translation of mRNA is closely related to YTHDF1, and the degradation of mRNA is closely related to YTHDF2, while YTHDF3 can regulate both the translation and degradation of mRNA [26]. Another protein that also promotes mRNA degradation is YTHDC2 [24]. YTHDC1, the only nuclear localization reading protein, can interact with multiple splicing factor proteins to regulate splicing of mRNA [6]. Although the various proteins of the YTH family act differently, they all share a common feature of recognizing and binding RNA in an M6A-dependent manner through their YTH domain [24]. The hnRNP family, without the YTH domain, can also recognize M6A-modified RNA in an indirect way. In a class of RNA recognized by the hnRNP family, M6A modifies as a switch, changing the structure of the mRNAs and promoting the RNA to be recognized by hnRNP family [3]. M6A reading proteins in this family mainly include hnRNPC, hnRNPG and hnRNPA2B1 [27]. hnRNPCs can bind to pre-mRNAs, regulate their stability, splicing, output, and translation [28]. hnRNPG regulates alternative splicing by interacting with RNAPII and pre-mRNA modified by N6-methyladenosine with its RGG motif to regulate alternative splicing [29], while hnRNPA2B1 regulates primary microRNA (miR) processing and alternative splicing by interacting with the microRNA microprocessor complex protein Dgcr8 [30]. IGF2BPs, which identify and bind to specific RNAs through the K-homologous (KH) domain and the RNA recognition motif (RRM) domain, recruit IGF2BP1-3 cofactors, including ELAV-like RNA-binding protein 1 (ELAVL1, also known as Hur) and Matrin 3 (MATR3) to mediate mRNA stability [3].

### M6A erasers

N6 methylation-modified RNA, in addition to acting after recognition by M6A reading proteins, can also be demethylated by demethylated proteins or proteins involved in demethylation, thereby reversing and regulating methylation-dependent biological processes. The most studied demethylases are fat-mass and obesity-associated protein (FTO) and α-ketoglutarate-dependent dioxygenase alkB homolog 5 (ALKBH5). Although FTO and ALKBH5 have similar catalytic structures, their mechanisms of action are entirely different [13]. FTO removes M6A modifications by demethylating 3-methylthymidine (3MT) in single-stranded DNA and 3-methyluracil (3 mU) in single-stranded RNA to produce two intermediates, N6-hydroxymethyladenosine (HM6A) and N6-formyladenosine (F6A) [25]. ALKBH5 removes M6A directly without producing any intermediates [14]. FTO and ALKBH5 also exhibited different tissue targeting. The study found that in mice, ALKBH5 was expressed most in the testes, while FTO expression was clustered in the brain [27]. FTO was originally discovered to target m6A in nuclear RNAs [31], while ALKBH5 targets cytosolic mRNA [25]. However, it has recently been shown that FTO is also present in the cytoplasm of certain cell types [31–33]. FTO preferentially targets the intron region in the pre-mRNA, thereby regulating alternative splicing of the pre-mRNA and processing of 3´UTR [25]. ALKBH5 regulates the nuclear output of specific RNAs and participates in splicing and stability of 3´UTR mRNAs [27].

## M6A and chronic kidney disease(CKD)

Chronic kidney disease is defined as chronic renal structural and functional dysfunction caused by various reasons, including various primary and secondary glomerulonephritis, tubular injury and renal vascular lesions, etc. In recent years, the incidence of chronic kidney disease (CKD) has been increasing globally. It is statistically estimated that the global burden of CKD among males aged 15–49 years had reached 1.94% in 2016 [34]. According to the World Health Organization, 14 out of every 100,000 people will die because of CKD in 2030 [35]. Therefore, it is urgent to explore the pathogenesis of CKD and to develop more effective treatments. Here, we summarize the relevant mechanisms involved in CKD caused by M6A modifications in recent years (Table 1).

**Table 1:** The mechanism of M6A in CKD and AKI

Kidney disease	Cell	Mechanism of injury	M6A regulators	Target	References
CKD	Podocyte	Inflammation	IGF2BP2, METTL3, METTL14	TIMP2, PI3K, Sirt1	[36–38]
		Apoptosis	METTL3, METTL14	TIMP2, Sirt1	[36, 38]
		Autophagy	METTL14	Sirt1	[38]
		Pyroptosis	YTHDF2, METT14, METTL3	lncRNA TINCR, PTEN	[39–41]
	MCs	Fibrosis	YTHDF1, METTL3	YAP, NSD2	[42, 43]
		Inflammation	METTL3	lincRNA-Gm4419	[44]
	GECs	Inflammation	METTL14	α-Klotho	[45]
AKI	TECs	Inflammation	IGF2BP1, WTAP, FTO METTL3, ALKBH5	NLRP3, PPAR-α, E2F1 TAB3, MALAT1, miR-21-5p,	[46–52]
		EMT	METTL14, FTO, METTL3 ALKBH5	PTEN, MALAT1 LncRNA Gas5	[53–55]
		Apoptosis	METTL3, METTl14, WTAP FTO, AlKBH5	miR-873-5p, Nrf2 Foxd1, lncRNA121686	[56–59]
		Ferroptosis	Unknown	mmu-miR-7212-5p, SAT1, ACSL4, NFE2L2	[60, 61]

### M6A and podocyte injury

The glomerular filtration barrier is a molecular filtration device that screens molecules based on their size, charge, and shape to strictly limit the filtration of macromolecules and ensure that proteins are not easily lost. Podocyte are an important component of the glomerular filtration barrier. [62]. Podocyte foot processes overlap each other to form intercellular connections attached to glomerular capillaries, and the gap septum formed is the main barrier to prevent protein loss [63]. Destruction of the slit septum structure caused by damage or loss of podocytes can lead to severe impairment of their filtration barrier function, leading to the production of proteinuria [62]. It is one of the important pathogenesis of various glomerular diseases [63]. A series of biological reactions involved in podocyte injury, such as oxidative stress, immunological damage, play a key role in kidney disease [64]. In recent years, the study of M6A modification in podocytes injury has received attention.

### M6A and inflammation in podocyte injury

As one of the mechanisms of cell damage, inflammation has been used as a bridge to study various cellular damage. Similarly, the role of various inflammatory pathways and inflammatory mediators in podocyte injury has also been widely concerned. Studies have shown that activation of Notch signaling is strongly associated with podocyte damage [65–67]. In IgA nephropathy, Notch1 is activated by Toll-like receptor 4 (TLR4) and mediates inflammatory damage of podocytes by activating NF-κB [65]. Sirt6 exerts pleiotropic protective effects on podocytes, including anti-inflammatory effects, by inhibiting the expression of Notch1 and Notch4 [68]. Tissue inhibitor of metalloproteinase 2 (TIMP2) has also been found to be involved in the inflammatory process of AKI [69, 70]. Recently, the role of M6A modification in inflammatory injury of podocytes has also been gradually discovered. In the development of diabetic nephropathy, with the assistance of IGF2BP2, METTL3-mediated M6A modification of TIMP2 is involved in inflammatory damage in podocytes by regulating Notch signaling [36]. The study found that the M6A modification of TIMP2 promoted its mRNA stability, which in turn promoted its activation of Notch signaling [36].

NLRP3 inflammasomes, which were initially studied more in immune cells, have recently been found to play an important role in the sterile inflammation of diabetic kidney disease (DKD) [71]. Under the stimuli of high glucose, ROS and AGEs, the activated NLRP3 inflammasome induces the pro-inflammatory cytokines IL-1β and IL-18 by activating caspase-1 [72, 73]. In recent years, Liu, Tu [37] found that an extract of *Abelmoschus manihot,* the total flavones of *Abelmoschus manihot* (TFA) can alleviate podocytes damage caused by high glucose by regulating the activation of NLRP3 inflammasomes. M6A modification plays an important role in the podo-protective role of TFA. TFA regulates the PI3K/Akt pathway through METTL3-dependent M6A modification, thereby inhibiting the activation of NLRP3 inflammasomes [37]. Sirt1*,* a NAD + -dependent deacetylase, can also exert a protective role in podocytes by inhibiting the activation of NLRP3 inflammasomes[74]. The expression level of Sirt1 is decreased in injured podocytes, and the specific mechanism of its downregulation has recently been investigated. Lu, Liu [38] found that knocking out podocytes METTL14 restored Sirt1's mRNA and protein levels. METTL14 levels were elevated in both Adriamycin (ADR) or db /db mice, as well as in injured podocytes. qRT-PCR analysis showed that silencing METTL14 in podocytes inhibited the expression of inflammatory mediators such as MCP-1, IL-6 and TNF-α in ADR-treated mice [38]. It can be seen that one of the mechanisms by which METTL14 aggravates podocytes damage through N6-methyladenosine-dependent Sirt1 downregulation is to promote inflammatory damage [38].

This series of related studies have shown that methyltransferases METTL3 and METTL4 can participate in mediating inflammatory damage in podocytes by regulating N6-methyladenosine modifications of certain mRNAs. These research results provide a new target for the treatment of glomerular diseases with podocytes inflammatory injury as the main pathological mechanism.

### M6A and apoptosis in podocyte injury

TIMP2 is not only involved in cellular inflammatory processes, but is also closely related to apoptosis [69, 70, 75]. In the human granuloma cell line KGN, TIMP2 is upregulated by LncRNA-LET to activate the Wnt/β-catenin and Notch signaling pathways, promoting apoptosis [75]. The M6A modification of TIMP2 mRNA mediated by METTL3 in podocyte injury in diabetic nephropathy not only promotes inflammatory damage, but also apoptosis [36]. Both knockdown METTL3 and TIMP2 could reduce the expression of Notch3 and Notch4, indicating that METLL3-mediated TIMP2 mRNA M6A modification exerts a pro-apoptosis effect through the Notch signaling pathway [36]. It can be seen that there is a close relationship between M6A modification and apoptosis in podocyte injury. In mice with ADR nephropathy, the expression of the anti-apoptotic gene BCL2 was reduced and the pro-apoptotic cleavage of caspase-3 was increased, which was reversed after deleting METTL14 in podocyte [38]. This process is achieved by METTL14-mediated Sirt1 M6A modification, which in turn affects Sirt1 mRNA stability [38].

### M6A and autophagy in podocyte injury

As a self-protection mechanism of cells, autophagy plays the role of clearing the portal through the activation of a series of autophagic proteins when the cells are subjected to different stimuli. This role of it allows it to play a non-negligible role in maintaining cellular homeostasis [76]. Similarly, autophagy is also one of the mechanisms by which the kidneys exert an adaptive response to various stress responses. Abnormal autophagy can also lead to the development of various kidney diseases [77]. Recently, *Sirt1*-mediated autophagy has received widespread attention in kidney disease. *Cordyceps cicadae (C. cicadae)* alleviates renal hypertension damage and fibrosis by regulating Sirt1/FOXO3a pathway [78]. p53/miR-155-5p/Sirt1 signaling axis-mediated autophagy involved in the occurrence and development of diabetic nephropathy [79]. Podocyte Sirt1-mediated autophagy has also been studied. In diabetic kidney injury, sodium-glucose cotransporter–2 (SGLT2) inhibitors exert renal protective effects by promoting enhanced autophagy in the Sirt1/HIF-2α signaling pathway [80]. The M6A modification of Sirt1 has also been found to affect autophagy in podocyte. Silencing METTL14 can upregulate the expression of Sirt1, thereby promoting autophagy of podocytes and alleviating podocyte damage in mice with ADR nephropathy [38].

### M6A and pyroptosis in podocyte injury

Pyroptosis is a programmed cell death whose classic trigger pathway is the inflammasome/caspase-1/gasdermin [81]. NLRP3 is an inflammasome that triggers this pathway [82]. NLRP3-mediated pyroptosis also plays an important role in podocyte injury. sC5b-9 inhibits miR-486A-3p by upregulating the long non-coding RNA Kcnq1ot1, thereby promoting NLRP3-mediated podocytes pyroptosis [83]. Gao, Ma [84] found that sialic acid precursor N-acetylmannosamine (ManNAc) can alleviate podocytes pyroptosis by inhibiting ROS/NLRP3 in a diabetic kidney injury model. The AMPK/mTORC1/NLRP3 signaling axis is also one of the signaling pathways that mediate pyroptosis of podocyte, and fucoidan (FPS), a class of sulfated carbohydrates found in brown marine algae and echinoderms, has been found to alleviate podocyte damage in diabetic nephropathy by modulating this pathway [85]. With the continuous disclosure of the role of M6A modification, more and more studies have found that M6A modification can regulate NLRP3-mediated pyroptosis through various signaling pathways [39, 40]. Under the synergistic effect of YTHDF2, METTL14 inhibits NLRP3-mediated pyroptosis by downregulating lncRNA TINCR, thereby alleviating diabetic cardiomyopathy [39]. Emodin, the main active component of rhubarb, alleviates LPS-induced pyroptosis in 1321N1 cells by regulating METTL3-mediated expression of NLRP3 [41]. The PI3K/Akt/GSK-3β signaling pathway regulated by the M6A modification of PTEN is the upstream pathway of NLRP3 [40]. In podocyte injury caused by high glucose, it was also found that METTL3-mediated PTEN M6A modification activates NLRP3 inflammasome through the PI3K/Akt signaling axis, triggering pyroptosis. TFA can inhibit the above processes, thereby alleviating the damage of podocytes [37].

## M6A and glomerular mesangial cells

Glomerular mesangial cells (MCs) are stromal cells that share a structurally supportive role with the mesangial stroma [86]. Under physiological conditions, mesangial cells can participate in maintaining the homeostasis of the extracellular stroma by secreting soluble factors [87]; When exposed to harmful stimuli, MCs are activated to overproliferate and secrete some harmful factors involved in pathological processes such as glomerular fibrosis [88]. In particular, the deposition of some immune complexes can cause damage to MCs, leading to the occurrence of immune diseases, such as lupus nephritis [89] and IgA nephropathy [87]. With the in-depth study of M6A modification, the role of M6A modification in mesangial cell injury has been continuously highlighted.

### M6A and fibrosis in MCs

Renal fibrosis refers to the pathological process of loss of normal tissue in the kidneys and abnormal accumulation of extracellular matrix. MCs are one of the main cells involved in renal fibrosis [88]. During renal fibrosis, mesangial cells proliferate abnormally and the matrix proteins they secrete continue to accumulate, a process also known as mesangial dilation [90]. Recently, the regulatory role of M6A modification in the participation of MCs in fibrosis has been revealed.

YES-associated protein (YAP), an important regulator of myofibroblast transformation, was found to be closely associated with renal fibrosis [42, 91]. YAP and transcriptional coactivator (TAZ) with PDZ-binding motifs can promote renal fibrosis by activating transforming growth factor β (TGF-β)-induced Smad2/3 signaling. While verteporfin, a potent YAP inhibitor can exert an antifibrotic effect by interfering with YAP/TAZ-TGF-b/Smad crosstalk [92]. Xu, Chen [93] found that the transcription factor KLF4 can not only promote its expression by binding to the YAP promoter, but also accelerate the degradation of LATS1 to promote the nuclear translocation of YAP and thus promote YAP-mediated renal fibrosis. The Piezo1-p38MAPK-YAP pathway has also been found to be involved in renal fibrosis progression in MCs [94]. Recently, studies have found that YAP-mediated renal fibrosis is regulated by M6A modification [42]. TGF-β is a major contributor to renal fibrosis, which can promote renal fibrosis through both Smad and non-Smad signaling pathways [95]. Xing, He [42] found that YTHDF1 expression was elevated in MCs cultured with TGF-β, and at the same time, the expression of the signature protein α-SMA in myofibroblasts was also increased, indicating that YTHDF1 in mesangial cells was involved in the renal fibrosis process. Follow-up studies have shown that YTHDF1 exerts a profibrotic effect by upregulating YAP expression [42]. The findings of this study not only enrich the upstream pathway of YAP to promote fibrosis, but also provide new and effective targets for anti-fibrosis.

Under high-glucose stimulation, continuous renal damage promotes various pathological changes such as epithelial-mesenchymal transition (EMT) and endothelial-mesenchymal transformation (EndoMT), which ultimately promote renal interstitial fibrosis. Interstitial fibrosis is an irreversible metabolic change in the late stages of diabetic nephropathy [96]. NSD2, one of the SET histone 3 lysine 36 (H3K36) methyltransferase members, not only performs epigenetic regulation, but is also an important regulator of epithelial-mesenchymal transition (EMT) [97, 98]. Recently, the M6A modification of NSD2 has been found to be involved in the regulation of renal interstitial fibrosis in diabetic nephropathy [43]. Serum levels of NSD2 and METTL3 in patients with diabetic nephropathy are significantly reduced. MeRIP-qPCR analysis, which is a technique that quantifies the enriched RNA by qPCR after enriching the RNA with methylation modifications using m6A antibody showed a significant decrease in M6A modification levels of NSD2 in high-glucose-treated mouse mesangial cells (SV40-MES-13) [43]. Overexpression of NSD2 not only alleviates interstitial fibrosis in kidney tissue of mice with diabetic nephropathy, but also reduces fibrosis-related markers in HG-treated SV40-MES-13 cells [43]. This study showed that METTL3 in mesangial cells can improve their mRNA stability by promoting NSD2 M6A modification with the participation of YTHDF1, reducing interstitial fibrosis and alleviating the progression of diabetic nephropathy [43]. The M6A modification of NSD2 may become an effective target for the treatment of diabetic nephropathy in the future.

### M6A and inflammation in MCs

The classic inflammatory signaling pathway, the NF-κB signaling pathway, has been widely shown to be involved in inflammatory damage of MCs [99, 100]. Based on the disclosure of NF-κB signaling pathway in mesangial cells, the alleviating effect of some compounds on inflammatory damage of MCs has also been discovered. Diphenyl diselenide (DPDS) improves LPS-induced mesangial cell inflammation by inhibiting the NFκB/MAPK pathway [101]; Inhibition of the SphK1/S1P2/NF-κB pathway by resveratrol (RSV) alleviates the inflammatory lesions of MCs [102]. However, the molecular mechanism involved in NF-κB upstream signaling in MCs inflammatory responses remains to be further studied. Yi, Peng [103] have found that lincRNA-Gm4419 is involved in the inflammatory response of MCs mediated by the NF-κB/NLRP3 inflammasome signaling pathway in a high-glucose environment. MeRIP-seq detection of LncRNA and GO and KEGG analysis found that the M6A modification of LncRNA may be involved in the NF-κB signaling pathway-mediated inflammatory response in MCs [44]. Knockout of the METTL3 gene in mouse mesangial cells (MMC or MMCs) significantly reduced M6A RNA methylation, pro-inflammatory cytokine IL6, and TNF-α levels, further confirming this possibility [44]. Combined analysis of MeRIP-seq and RNA-seq to assess the alterations in the epitranscriptome-wide M6A profile of mouse MMCs induced by LPS also found that multiple differential genes obtained were involved in the inflammatory response of MCs [104]. This provides a reliable basis for exploring the more upstream molecular mechanism of MCs inflammatory response.

### M6A and glomerular endothelial cells injury

Glomerular endothelial cells (GECs), an abnormally flat cell [105] covering the luminal surface of the glomerular capillary, together with podocytes and basement membranes, form the glomerular filtration barrier [106]. GECs are highly fenestrated, with a large fenestrated area accounting for 20–50% of the entire endothelial cell surface area, which is necessary for the glomerulus to maintain permeability and perform a large number of filtration [105]. The glomerular endothelial cell lining is a negatively charged glycan calyx [107]. Its high charge barrier effect allows it to play a vital role in maintaining the homeostasis of glomerular filtration [106]. In addition, glycocalyx can also regulate vascular permeability, maintain fluid balance, and inhibit the adhesion of cells and platelets to endothelial cells [107]. It can be seen that endothelial cells play a non-negligible role in the glomerulus. To further explore the pathological mechanism of glomerular-related diseases, it is necessary to fully understand the mechanism of glomerular endothelial cell damage.

A receptor for fibroblast growth factor-23 (FGF-23), α-Klotho is a monotransmembrane anti-aging protein [108] whose role in kidney disease has been extensively studied [109, 110]. As a kidney protective molecule, α-Klotho can not only ameliorate renal fibrosis and delay the progression of CKD by inhibiting the Wnt/β-catenin signaling pathway in renal tubular epithelial cells [111], but also alleviate diabetic nephropathy by promoting tubular autophagy through the AMPK and ERK pathways [112]. Recently, the role of α-Klotho in the progression of diabetic nephropathy has been further studied in glomerular endothelial cell injury [45]. Li, Deng [45] found that overexpression of METTL14 increased TNF-α, IL-6 and ROS levels promoting glomerular endothelial cell injury. In vivo studies found that treatment of db/db mice with METTL14-expressing rAAV resulted in increased 24-h urine protein and kidney weight, among others, and decreased body weight. However, overexpression of α-klotho reversed all of these changes. Increased levels of M6A modification of Klotho detected by RNA immunoprecipitation PCR (RIP-qPCR), a technique for quantifying RNAs that bind to target proteins suggest that METTL14 promotes glomerular endothelial cell injury by increasing N6 methylation modification of α-klotho and thus downregulating α-klotho expression in a high-glucose environment [45]. This study not only revealed the nephroprotective effect of α-klotho but also provided a new direction in the study of glomerular endothelial cell injury, i.e., M6A modification. The role of M6A modification in kidney injury has been widely uncovered, but its role in glomerular endothelial cell injury is poorly studied and still needs to be explored.

## M6A and acute kidney injury (AKI)

Acute kidney injury (AKI) is a group of clinical syndromes characterized by a sudden (within 1–7 d) and sustained (> 24 h) decline in renal function. Currently, both AKI and CKD are important factors leading to end-stage renal disease. It is estimated that 2 million people died of AKI in 2013 [35]. The global burden of AKI has compelled us to accelerate the exploration and understanding of it. Similarly, M6A modifications are involved in AKI, especially in renal tubular epithelial cells (Table 1).

### M6A and tubular epithelial cell injury

As the most abundant cell type in the kidney, tubular epithelial cells (TECs) are the main cell types that drive high metabolism in the kidney, and they are responsible for renal transport activity and reabsorption [113]. As the main component of the kidneys, the renal tubules are highly susceptible to damage. In various damages, TECs drive a series of pathological processes by synthesizing and secreting various active substances, aggravating kidney damage [114]. Therefore, a deeper understanding of the pathological process and mechanism involved in tubular epithelial cell injury is essential to study renal injury.

### M6A and inflammation in TECs

As mentioned previously, the impacts on inflammatory damage of podocytes by NLRP3 inflammasome regulated by N6 methylation modifications have been reported [37, 85]. Related research has also indicated that the inflammatory response mediated by NLRP3 inflammasome in renal tubular epithelial cell injury is similarly regulated by M6A modifications [46, 47]. Lan, Xu [46] discovered that WTAP, one of the M6A writers, was abundantly expressed in patients with DN and in HG‑induced HK‑2 cells and positively associated with the release of pro-inflammatory cytokines in HK-2 cells. Further studies revealed that NLRP3 was the target of WTAP-mediated M6A modification. With the engagement of IGF2BP1, WTAP influences the expression of NLRP3 mRNA by regulating its stability through M6A modification, which in turn promotes the inflammatory response of HK-2 cells [46]. Yu, Hu [47] found that activation of NLRP3 inflammasome was also present in inflammatory kidney injury caused by alcohol. FTO expression was reduced in both kidney and HK-2 cells after alcohol stimulation, and whole-genome methylation sequencing revealed that alcohol stimulation caused methylation of FTO, which in turn inhibited its expression [47]. Methylated RIP-qPCR (MeRIP-qPCR) results showed that PPAR-α M6A mRNA levels were increased after alcohol stimulation and decreased after overexpression of FTO [47]. PPAR-α inhibitor, MK-886 promotes activation of NLRP3 inflammasome and NF-κB signaling pathways and release of pro-inflammatory cytokines (TNF-α, MCP-1, IL-6, IL-1β, and IL-33) [47]. This suggests that alcohol enhances PPAR-α M6A modification by inhibiting FTO expression and thereby activates NF-κB/NLRP3 inflammatory vesicles to promote inflammatory kidney injury.

Infectious acute kidney injury (AKI) is characterized by inflammation [48]. Macrophage movement inhibitory factor (MIF) serves as an upstream inflammatory factor that performs a crucial role in the pathology of acute kidney injury [115]. E2F1 was found to promote MIF expression as a transcription factor. The presence of an M6A recognition site in the 3'-UTR region of the E2F1 gene and further actinomycin D experiments revealed that the stability of E2F1 mRNA was influenced by IGF2BP1-mediated M6A modifications [48]. IGF2BP1 is mainly localized in renal TECs [48]; thus, the IGF2BP1-mediated E2F1/MIF pathway in renal TECs may be a valuable target to mitigate septic acute kidney injury. The role of M6A modifications in acute kidney injury is continuously being revealed. Another study found increased expression of METTL3 in renal TECs in acute kidney injury [49]. Further studies revealed that METTL3 exerts a pro-inflammatory effect in the inflammatory responses induced by TNF-α, cisplatin and LPS in HK2 cells and mouse TEC [49]. TAB3, a member of the TAK1 binding proteins, plays a crucial role in the inflammatory response [116]. Methylated RNA immunoprecipitation sequencing (MeRIP-seq) and correlation analysis show that TAB3 is a direct target of METTL3-mediated inflammatory response in renal TECs. METTL3 enhances TAB3 mRNA stability by mediating M6A modification of TAB3 to promote IGF2BP1 binding to TAB3 [49]. These studies suggest that the METTL3/TAB3 axis in renal TECs may be a potential therapeutic target for the treatment of acute kidney injury.

DROSHA/DgCr8 and DICER-mediated enzymatic reactions are the first step in the biogenesis of miRNAs [50, 117]. M6A modifications have been shown to be present on more than 200 miRNAs and some of them have been shown to play an integral role in the initiation of miRNA biogenesis [117]. The most common M6A writer, METTL3, is involved in the initiation of miRNA biogenesis via methylation to label Pri-miRNAs to be recognized and bound by Dgcr8 [50]. M6A modifications are not only involved in the maturation of miRNAs, but also perform essential roles in their physiological functions and the pathological mechanisms involved. miR-21 has been found to be involved in the process of pulmonary fibrosis [118]. The miR-21-5p/SPRY1/ERK/NF-kB signaling axis has recently been confirmed to promote the progression of obstructive renal fibrosis through an inflammatory response in a mouse model of unilateral ureteral obstruction (UUO) [51]. Further studies in UUO mice and HK-2 cells reveal that METTL3-mediated M6A modification drives the pro-inflammatory effects of miR-21-5p and exacerbates obstructive renal fibrosis by promoting its maturation [51]. Thus, we should not only focus on the contribution of M6A modification of mRNAs, but also on the role of M6A modification of non-coding RNAs in kidney diseases.

With the understanding of the pathophysiological mechanisms of M6A modification in inflammatory injury of renal TECs, it is increasingly certain that M6A modification is a potential therapeutic target for the treatment of a number of renal diseases. Recently, studies have begun to focus on and validate the role of M6A modification in the pharmacological mechanisms of some drugs. Dexmedetomidine is a highly selective α_2_-adrenergic agonist with effects such as sedation and analgesia [119]. Dexmedetomidine has been shown to inhibit the inflammatory response in ischemia–reperfusion kidney injury [120]. Zhu and Lu [52] found that dexmedetomidine was able to inhibit the expression of pro-inflammatory cytokines such as TNF-α, IL-6 and IL-1β in LPS-induced HK-2 cells by blocking ALKBH5. Further studies identified MALAT1 as a direct target of ALKBH5 demethylation modification. The anti-inflammatory effect of dexmedetomidine through inhibition of the ALKBH5/MALAT1 axis is sufficient to demonstrate its potential in the prevention and treatment of renal injury in sepsis [52].

### M6A and epithelial-mesenchymal transformation in TECs

The process by which epithelial cells lose their junctions and apical-basal polarity and reorganize their cytoskeleton into mesenchymal cells is called epithelial-mesenchymal transition. The process is usually accompanied by a decrease in the expression of epithelial proteins such as E-calmodulin and an upregulation of mesenchymal marker proteins such as vimentin and fibronectin [121, 122]. EMT in the kidney is the process by which renal TECs are converted to fibroblasts by changing their phenotypic characteristics to mesenchymal. And EMT is one of the mechanisms of renal fibrosis. [121]. Studies have revealed that EMT occurs predominantly in the proximal part of the kidney and has become a critical factor in the abnormal functioning of the renal unit in chronic kidney disease caused by diabetes [123]. Fully grasping the effect of M6A modification on the EMT process in renal TECs is a prerequisite for the study of renal fibrosis-related diseases.

HDAC5, a member of the histone deacetylase family, has been identified to be associated with the EMT process in some cancer cells [124, 125]. PI3K/AKT signaling pathway-regulated HDAC5 was also revealed to facilitate the EMT process in high-glucose-stimulated HK-2 cells [53]. PTEN was known to be a suppressor of the PI3K/AKT signaling pathway [126]. Further studies revealed that M6A modifications are also involved in this process. METTL14 affects HDAC5-mediated EMT in renal TECs with diabetic nephropathy by regulating the PI3K/Akt signaling pathway through M6A modifications of PTEN [53]. This suggests that M6A modification exerts a non-negligible effect on the EMT process in renal TECs in the context of diabetes.

Non-coding RNAs are a class of RNAs that do not have the ability to code for proteins, but their regulatory role in cellular physiological functions and related diseases should not be underestimated. Long-stranded non-coding RNAs (LncRNAs), a type of non-coding RNA over 200 nucleotides in length, have been found to be involved in the fibrotic process in a variety of organs [127]. An increasing number of studies demonstrate that the function of LncRNAs in a variety of diseases is regulated by M6A modifications [128, 129]. Recently, it has also been shown that some LncRNAs in the kidney are regulated by M6A modifications and are involved in the renal EMT process [54, 55]. Li [54] found that FTO was upregulated and LncRNA Gas5 was downregulated in an in vitro renal interstitial fibrosis (RIF) model and that both were jointly involved in TGF-β1-induced EMT in HK-2 cells. The role of the removal of M6A modification of lncRNA Gas5 mediated by FTO in renal EMT may be a therapeutic target for future alleviation of renal interstitial fibrosis. As one of the early disease-associated LncRNAs discovered, MALAT1 has been widely studied in a range of tumors [130]. Recently, Liu, Zhang [55] found that MALAT1 is involved in TGF-β1-induced renal EMT in vitro and in vivo by sponging miR-145 and regulating FAK expression. Further studies in HK2 cells showed that METTL3-mediated M6A modification participates in the EMT process through the MALAT1/miR-145/FAK signaling pathway. This study provides an experimental basis and potential therapeutic target for renal fibrosis in obstructive nephropathy [55]. It can be seen that the role of M6A modification-mediated EMT in obstructive nephropathy should be emphasized in treatment-related studies. At present, studies have shown that genistein inhibits the EMT of obstructive nephropathy by upregulating the expression of ALKBH5 and reducing the level of M6A modification [131].

### M6A and apoptosis in TECs

Colistin is a concentration-dependent bactericidal antibiotic that is extremely effective against multi-resistant Gram-negative bacteria, but its nephrotoxicity greatly limits its application in clinical situations [132]. Keap1/Nrf2, an important cytoprotective signaling pathway against oxidative stress [133], has been revealed as one of the mechanisms by which colistin mediates nephrotoxicity. And METTL3-mediated M6A modification is the upstream mechanism of this pathway mediating nephrotoxicity [56, 134]. In colistin-induced kidney injury, M6A modification is involved in oxidative stress-mediated apoptosis through regulation of the Keap1/Nrf2 signaling pathway. Pathological manifestations include dilatation and structural alterations of the renal tubules, with apoptotic morphological changes such as nuclear consolidation and chromatin edge aggregation visible on electron microscopy [134]. Wang, Ishfaq [56] further demonstrated in renal TECs that METTL3-mediated M6A modification is involved in colistin-mediated apoptosis by regulating the maturation of miR-873-5p. It can be seen that METTL3-mediated M6A modification may be one of the important targets to alleviate colistin nephrotoxicity. The method of inhibiting the METTL3/M6A/miRNA-873-5p/Keap1/Nrf2 signaling pathway can effectively broaden the application of colistin.

Cadmium is a heavy metal contaminant that can cause damage to the kidneys when it enters the body. And it accumulates mainly in the proximal tubular cells [135]. After treatment of HK-2 cells with cadmium sulfate, increased levels of ROS and an apoptotic morphology were found. Real-time quantitative polymerase chain reaction (RT-PCR) detected changes in the expression of M6A regulatory protein. Further studies revealed that M6A modification also promotes apoptosis in cadmium-induced nephrotoxicity via the Nrf2 pathway [57].

Cisplatin is a chemotherapy drug widely used in various tumors. However, its clinical application is limited by its various side effects, including kidney damage [136]. Due to the pharmacokinetic profile of cisplatin, it accumulates in the kidneys, especially in TECs. Excessively high drug concentrations promote oxidation and activate the apoptosis process [137, 138]. Elevated levels of total renal M6A were detected in cisplatin-induced acute kidney injury (cis-AKI) in mice, accompanied by alterations in METTL3, Mettl14, WTAP, FTO and AlKBH5. The gene microarray method suggested that a total of 618 mRNAs and 98 LncRNAs were significantly differentially methylated after cisplatin treatment [138]. Zhou, Wu [58] further demonstrated reduced FTO expression and increased M6A levels after cisplatin treatment in HK2 cells. Meclofenamic acid (MA), a pharmaceutical compound targeting FTO, increases M6A levels, promotes cisplatin-induced apoptosis and is involved in the development and progression of cis-AKI [58].

Foxd1, first identified in forebrain neuroepithelial cells, is a forkhead transcription factor that plays an important role in biological processes such as kidney and retinal development [139]. Recently, the role of mRNA M6A modification levels of Foxd1 in renal ischemia–reperfusion injury (IRI) has been demonstrated in the establishment of hypoxia/reoxygenation (H/R) cell models by NRK-52E cells and in IRI mouse models. Increased expression of METTL3 in IRI and H/R models, involved in M6A modification of Foxd1. The mechanism of METTL3/M6A/Foxd1 in IRI is mainly to promote apoptosis [140]. Similarly, Pan, Xie [59] found that MMU-lncRNA 121686 mediated ischemia–reperfusion-induced apoptosis in proximal tubular cells (BUMPT) and HK-2 cells by sponging miR-328-5p and upregulating HtrA3. MMU-lncRNA 121686 is positively and directly regulated by METTL3. And silencing METTL3 significantly attenuated ischemia, sepsis and vancomycin (Van) induced AKI [59]. The above studies suggest that METTL3-mediated M6A modifications play a non-negligible role in IRI. And the role of other M6A-related regulatory proteins should also be taken into account.

### M6A and ferroptosis in TECs

Ferroptosis, one of the classes of cell death, refers to the activation of oxidative stress by affecting glutathione peroxidase, which in turn leads to cell death. The process is usually accompanied by the intracellular buildup of iron and lipid peroxidation [141]. Increasingly, studies have shown that ferroptosis exists in the tubular epithelium of a wide range of renal diseases [142, 143]. A series of high-throughput sequencing analyses and pathway enrichment analyses revealed that in sepsis-associated acute kidney injury (SA-AKI), the pathway with the highest enrichment score was ferroptosis. Further studies in mouse renal TECs (TCMK-1) revealed that the mmu-miR-7212-5p-Hmox1 axis exerts an essential effect in ferroptosis, while this signaling axis is regulated by M6A modifications [60]. Similarly, Ni, Bai [61] performed an analysis of the correlation between M6A-related genes and ferroptosis associated genes (FAGs) in acute kidney injury (AKI). The results showed that FAGs (SAT1, ACSL4 and NFE2L2) were not only expressed at increased levels in AKI but also positively correlated with M6A methylation gene expression levels. Among them, NFE2L2 was of more diagnostic value [61]. Although the above studies suggest a non-negligible role for M6A modification in ferroptosis in renal TECs, more specific mechanisms remain to be explored.

## M6A and renal cell carcinoma

Renal cell carcinoma (RCC), which accounts for 2–3% of malignancies in adults, is a predominantly male disease. It is the seventh most common cancer in men [144]. Approximately 73,750 new cases and 14,830 deaths in the USA in 2020 [145]. There are three main subtypes of renal cell carcinoma: the most common and most aggressive renal clear cell carcinoma (ccRCC) (65–70%), papillary renal cell carcinoma (PRCC) (15–20%), and the least aggressive suspicious renal cell carcinoma (ChRCC) (5–10%) [146]. Although molecularly targeted therapies have been widely used in the clinic and surgical treatment continues to advance, the survival rate of patients with renal cell carcinoma is still not promising. Therefore, further exploration of the pathogenesis, biomarkers and therapeutic targets of renal cell carcinoma is still urgently needed [147].

**Fig.2:**
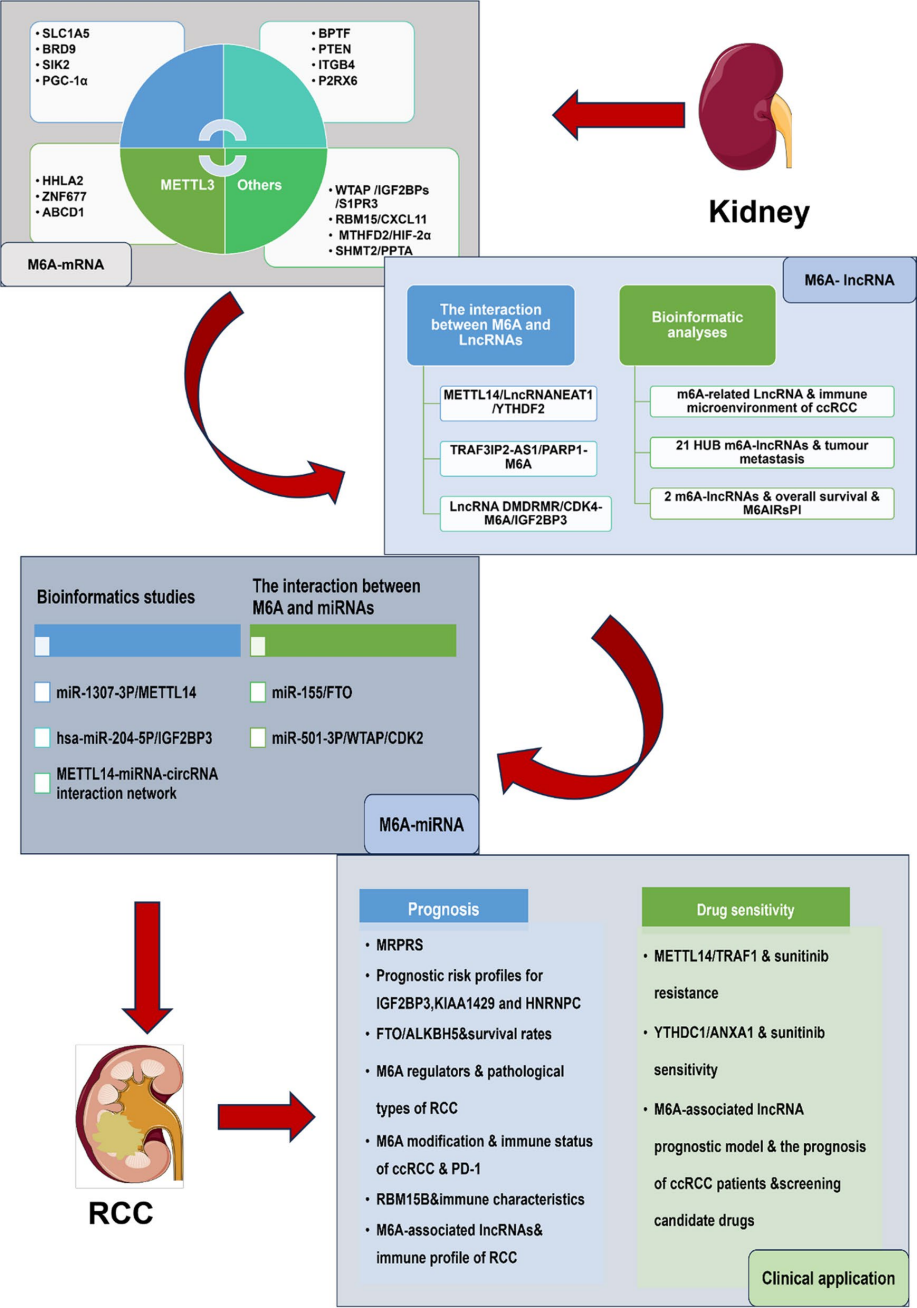
The role of M6A modification in RCC. M6A is involved in the development of RCC by interacting with different mRNAs, LncRNAs and miRNAs, and thus has been found to be useful in the clinical treatment and prognosis of RCC

As one of the most common post-transcriptional modifications, M6A modifications have been found to be involved in various tumor stages anyway and are expected to be effective anti-cancer targets [148]. Similarly, its role in renal cell carcinoma continues to be revealed [145, 147, 149]. Chen, Zhou [150] established the first M6A whole transcript profile of human renal cell carcinoma using high-throughput sequencing technology combined with bioinformatics analysis to describe the M6A modification patterns in renal cell carcinoma and normal tissues. Li, Hu [151] identified for the first time two M6A modification patterns in renal clear cell carcinoma (KIRC) and developed an M6A scoring algorithm to quantify individual M6A modification patterns. The identification of M6A clusters and M6A scores is significant for the elucidation of the immunophenotype, prognosis and prediction of immunotherapeutic response in KIRC. Based on the above bioinformatics studies, the involvement of M6A modifications in the specific pathological mechanisms of renal cell carcinoma has also been continuously disclosed (Fig. 2).

### M6A and mRNA in renal cell carcinoma (RCC)

Although M6A modifications are present in a variety of RNAs, the M6A modifications are the most common modifications in mRNA [147]. M6A modifications of mRNA have also been increasingly studied in renal cell carcinoma in recent years [152, 153]. Increasingly, studies have identified the involvement of demethylase FTO in the development of renal cell carcinogenesis by demethylation modification of multiple mRNAs [154–156]. Clear cell carcinoma of the kidney, the most prevalent form of renal cell carcinoma, whose main feature is the deletion of the von Hippel-Lindau (VHL) oncogene [157]. Inactivation of VHL usually leads to activation of the hypoxia-inducible factors HIF-1 and HIF-2 and their downstream targets, thereby promoting ccRCC [156]. *HIF-2* was the most significant driving factor in *ccRCC* progression. As a resul*t,* inhibitors of HIF2 has a better therapeutic effect at the in vitro level of *ccRCC* as *compared* *to* anti‐angiogenic drugs [157]. However, in ccRCC with low HIF-2 expression, the inhibitor loses its anticancer effect. Finding HIF-independent anti-cancer targets becomes essential, while FTO is a potential anti-cancer HIF independent target for ccRCC [156, 157]. On the one hand, FTO promotes metabolic reprogramming and survival of VHL-deficient ccRCC cells by targeting SLC1A5 [156]. On the other hand, FTO promotes the progression of HIF-2α-deficient (HIF-2α^low/−^) ccRCC by demethylating BRD9 [157].

Salt-inducible protein kinase (SIK) is a serine/threonine protein kinase. SIK2, a member of the SIK family, has been found to exert an important contribution to the formation of autophagic vesicles and autophagy [158]. Recently, SIK2-mediated autophagy has also been found to be involved in the progression of RCC. Xu, Zhou [155] found that the stability of SIK2 mRNA was regulated by FTO-mediated demethylation modification and that FTO/SIK2 promoted ccRCC progression through mediated autophagy. However, the role of FTO in renal cells is controversial. Zhao et al. summarized the studies of FTO in RCC in the past few years and found that six showed that FTO had tumor suppressive effects and three showed that it had carcinogenic effects [154]. Zhuang, Zhuang [159] then found that the expression of FTO was suppressed in ccRCC. Further studies found that in VHL-deficient ccRCC, FTO promoted the expression of PGC-1α through demethylation modification, restored mitochondrial activity and inhibited tumor growth. Although the role of FTO in renal cell carcinoma has been widely noted, further studies are needed to explain the controversy and to determine its role in this disease.

Tumor cells meet their altered biosynthetic, bioenergetic and redox requirements by altering their nutrient uptake and metabolic pathways. This process is known as metabolic reprogramming. Metabolic reprogramming in renal cell carcinoma is mainly associated with VHL deletion and Ras-PI3K-AKT-mTOR pathway activation [160]. Negative regulation of METTL14-mediated BPTF in RCC is involved in cellular glycolytic reprogramming and drives lung metastasis [161]. PI3K/AKT signaling pathway may be one of the pathways involved in RCC for METTL14 [162, 163]. Downregulated METTL14 in RCC promotes proliferation and migration of renal cell carcinoma cells by inhibiting PTEN expression through M6A modification. The PI3K/AKT signaling pathway is a downstream pathway of PTEN in this process [162]. However, Liu, Sun [163] found that METTL14 negatively regulates ITGB4 and thus stimulates EMT processes and PI3K/AKT signaling in RCC. This pathway is also involved in the metastasis of RCC. Furthermore, it has been shown that P2RX6, one of the receptors for ATP, is similarly regulated by METTL14-mediated M6A modification and is involved in the invasion and metastasis of kidney cancer cells. Nevertheless, the downstream pathway in which it functions is the Ca ^(2+)^-p-ERK1/2-MMP9 signaling pathway [164]. In summary, the role of METTL14 in the migration and proliferation of RCC cannot be ignored. The future development of anti-tumor drugs targeting METTL14 may bring benefits to the majority of RCC patients.

Human endogenous retroviral H long terminal repeat sequence-associated protein 2 (HHLA2), a member of the B7 family, is highly expressed in many cancers. High expression of HHLA2 has been shown to be negatively correlated with overall survival in patients with hepatocellular carcinoma and positively correlated with lymphatic metastasis of certain cancers [165]. HHLA2 has also been found to be involved in the onset and development of RCC, while METTL3 acted as its upstream signaling molecule by mediating M6A modifications to enhance its mRNA stability [152]. METTL3/HHLA2 may be a new target for future RCC immunotherapy. Cell cycle protein-dependent kinase inhibitor 3 (CDKN3) is an important regulatory protein of the cell division process. Studies have shown that high expression of CDKN3 significantly promotes RCC cell proliferation and resists apoptosis [166]. Recently, Li, Cao [167] showed that the transcription factor ZNF677 can inhibit the transcription of CDKN3 by binding to its promoter region to exert anti-cancer effects. To further explore the factors regulating ZNF677, the CRISPR/dCas13b-METLL3 system was used for targeted methylation of ZNF677. It was found that METTL3 not only enhanced the stability of ZNF677 mRNA with the involvement of IGF2BP2, but also promoted the translation of ZNF677 in a YTHDF1-dependent manner [167]. This shows that the effect of M6A modification on mRNA affects both its stability and the efficiency of its translation. Shi, Dou [168] then found that translation of ABCD1 is regulated by METTL3-mediated M6A modification. And the pro-carcinogenic role of ABCD1 in RCC was further enhanced. Therefore, we should also focus on the effect of M6A modification on mRNA translation in our subsequent studies.

In addition to the common M6A regulatory proteins mentioned above, the role of other regulatory proteins in RCC has also been reported. WTAP and IGF2BPs enhance the stability of S1PR3 mRNA to promote its expression and contribute to RCC via the PI3K/AKT pathway [153]. Oncogenes widely present in a variety of tumors, RBM15 also regulates CXCL11 expression in an M6A-modified manner in ccRCC. Further studies revealed that the RBM15/CXCL11 axis significantly promoted the growth, metastasis and macrophage invasion of renal clear cell carcinoma [169]. A mitochondrial enzyme involved in one-carbon metabolism, MTHFD2 promotes glycolysis in RCC by regulating the M6A modification of HIF-2α. The positive feed-forward loop formed by MTHFD2 and HIF-2α exerts an important effect in tumor metabolic reprogramming and growth [170]. Serine hydroxy methyltransferase 2 (SHMT2) is the rate-limiting enzyme for serine/glycine biosynthesis and one-carbon metabolism. It increases M6A levels in RCC via the endogenous methyl donor SAM. PPTA involved in RCC cell proliferation is regulated by SHMT2 in an M6A-IGF2BP2-dependent manner with a catalytic serine/glycine switch [171].

### M6A and LncRNA in renal cell carcinoma (RCC)

As mentioned above, in addition to M6A modifications of mRNAs involved in the progression of kidney disease, M6A modifications of some non-coding RNAs also serve an overarching effect. Interestingly, there is an interaction between M6A modifications and non-coding RNAs in neoplastic diseases. M6A modifications exert a regulatory effect on non-coding RNAs. Abnormalities in non-coding RNA levels can also affect M6A levels [172]. LncRNA NEAT1 is tagged by METTL14-mediated M6A modifications in RCC. YTHDF2 promotes the degradation of LncRNA NEAT1 by selectively recognizing the M6A modification on NEAT1 and thus exerts anti-cancer effects [173]. However, it has been shown that LncRNAs can be involved in RCC progression by regulating the level of M6A modifications [174, 175]. TRAF3IP2AS1, a member of the tumor necrosis factor receptor-associated proteins, is a natural antisense lncRNA expressed on the basis of TRAF3IP2. Overexpression of TRAF3IP2-AS1 promotes the decline of PARP1 mRNA through M6A modification. Thus, it can exert tumor suppressive effects in NONO-TFE3 tRCC, a novel RCC isoform [174]. Similarly, LncRNA DMDRMR regulates the cell cycle protein CDK4 in an M6A modification-dependent manner in RCC. On this basis, it facilitates the cellular G1/S transition in synergy with IGF2BP3, which in turn promotes cell proliferation [175].

In addition to some of the above mechanistic studies of LncRNA and M6A modifications in RCC, some bioinformatic analyses have uncovered their potential clinical significance. Liu, Zhuang [176] identified M6A-related LncRNA s as a possible key regulator in the immune microenvironment of ccRCC by high-throughput bioinformatics and statistical analysis. In addition, ccRCC data from the TCGA database were analyzed by WGCNA to identify 21 HUB M6A-LncRNAs associated with tumor metastasis. 2 M6A-LncRNAs associated with overall survival were selected from the 21 to construct and validate the M6A-LncRNAs prognostic index (M6AlRsPI). In this study, the ceRNA network constructed from 21 HUB M6A-LncRNAsg showed well potential oncogenic regulatory pathways. M6AlRsPI and molecular signatures of two M6A-LncRNAs were fully analyzed to investigate the potential modulatory processes in KIRC [177]. Correspondingly, Yu, Mao [178] constructed and validated an M6A-related LncRNAs prognostic marker based on the TCGA database, which can accurately predict the prognosis of KIRC patients.

### M6A and microRNA (miRNAs) in renal cell carcinoma (RCC)

MicroRNAs (miRNAs) are highly conserved, short non-coding RNA families with long half-lives, whose role in RCC has been extensively studied [179]. Yu, Liu [180] constructed expression profiles of RNA M6A regulators in 13 cancer types using data from TCGA. The expression changes of M6A regulators regulated by miRNA in pan-cancer were further analyzed. Three miRNA/mRNA were found to have possible oncogenic roles in ccRCC, and two of them (hsa-miR-1307-3p/METTL14 and hsa-miR-204-5p/IGF2BP3 were validated. Another non-coding RNA, circRNA, has recently been identified as being involved in the progression of RCC. circPOLR2A, under the regulation of YTHDF2, activates the ERK pathway by regulating UBE3C-mediated ubiquitination and degradation of PEBP1 protein, which significantly contributes to the metastasis of RCC [181]. As mentioned earlier, M6A modification in RCC not only regulates non-coding RNAs, but is also regulated by non-coding RNAs. In addition to being regulated by M6A modifications, circRNAs can also control M6A modifications by regulating M6A regulatory proteins. Wang, Zhang [182]. determined the localization of METTL14 in ccRCC tissues with human protein mapping, analyzed the major MiRNAs associated with ccRCC with OncoLnc and Starbase, and predicted the corresponding CircRNAs interacting with miRNAs by CircBank. The METTL14-miRNA-CircRNA interaction network was constructed based on the results of the above analysis. In addition, this study suggests that circRNAs may act as miRNAs sponges regulating METTL14 mRNA, thereby affecting the progression of KIRC [182]. Taken together, miRNAs are the mediator of circRNA regulating M6A modifications.

In addition to the above bioinformatics studies, specific miRNAs interacting with M6A in RCC have been reported. This provides potential targets for future clinical treatment of RCC. miR-155 decreases FTO protein levels and increases M6A levels in ccRCC by binding directly to the FTO mRNA 3'UTR [183]. Flow cytometry and CCK8 assays revealed that miR-501-3p could inhibit the proliferation of kidney cancer cells. Overexpression or knockdown of miR-501-3p was accompanied by variations in N6-methyladenosine (M6A) levels. Further studies revealed that WTAP, a target of miR-501-3p, is involved in regulating the progression of RCC by modulating CDK2 [184]. This study not only gives us a new understanding of the role of miRNAs in RCC, but also provides a more detailed target for future treatment of RCC.

## The clinical application of M6A in renal injury

### The potential treatment of M6A modification in renal diseases

The development of high-throughput sequencing technologies has driven the study of M6A modifications in diseases, which in turn has facilitated the exploration of the potential contribution of M6A modifications in disease treatment. Studies have been conducted to explore M6A-targeted anticancer drugs through traditional drug-like natural product screening as well as artificial intelligence and chemical synthesis [148]. Although potential renal disease drugs targeting M6A modifications have not been systematically screened and developed, there are studies in this area. Wang, Wang [185] found that genetic and pharmacological inhibition of METTL3 can significantly reduce renal inflammatory injury and is a potential treatment for AKI. The small molecule compound Cpd564 was virtually screened by high-throughput sequencing and further validated to bind and inhibit METTL3 activity, which could further attenuate renal inflammatory injury [186]. The anticancer effects of various flavonoids have been revealed [148]. The total flavones of *Abelmoschus manihot* (TFA), the main components of Huangkui capsule (HKC; the local name in China) attenuates podocyte inflammatory injury by targeting METTL3 and is a potential drug for the treatment of DKD [37]. Genistein [4, 5, 7-trihydroxyisoflavone], a soy isoflavone can regulate EMT to attenuate renal fibrosis by up-regulating ALKBH5 and is a potential drug for CKD treatment [131]. However, Zhu et al. found that the inhibitory effect of dextromethorphan on ALKBH5 attenuates LPS-induced HK-2 cell injury and is a potential target for sepsis-induced kidney injury [52]. The level of ALKBH5 expression in the kidney that can be maintained in order to maintain normal renal physiological function still needs to be further explored. In addition, many other compounds have been found to exert anticancer effects by modulating M6A such as phenols, alkaloids, anthraquinone and terpenoids [148]. Perhaps these compounds also play an important role in kidney injury and are potential drugs to alleviate kidney injury, which requires further exploration.

### The clinical application of M6A in renal cell carcinoma (RCC)

All research on pathogenesis becomes valuable only if it is given clinical significance. Based on the above studies, the application of bioinformatics gives clinical significance to the study of M6A modifications in RCC. By collecting data from TCGA, combined with statistical analysis, the investigators not only identified aberrantly expressed M6A regulators in ccRCC, but also uncovered significant relationships between them and clinical features and established risk profiles to predict prognosis of ccRCC [187, 188]. The M6A regulator prognostic risk score (MRPRS) was constructed by Yu, Liu [180] based on cohort studies, sequencing data analysis, and several bioinformatics methods. The MRPRS is of great importance in predicting clinical outcomes and treatment response in ccRCC patients. Having understood the value of M6A in predicting the prognosis of RCC, a number of studies have been devoted to exploring the clinical significance of certain specific M6A regulators. Strick, von Hagen [189] identified aberrant expression of FTO and ALKBH5 in RCC by qRT-PCR and tissue microarray techniques, and found that reduced mRNA levels of ALKBH5 and FTO were associated with lower survival rates in RCC patients. It also demonstrated that ALKBH5 and FTO could be used as biomarkers to assess the prognosis of RCC patients. Prognostic risk profiles for IGF2BP3, KIAA1429 and HNRNPC have been successfully constructed to accurately predict survival outcomes in patients with papillary renal cell carcinoma (pRCC) [190, 191]. M6A regulators are not only aberrantly expressed in RCC, but also differ between various pathological types of RCC. Further bioinformatic studies suggest that M6A regulators are associated with malignant progression of RCC and are potentially valuable for prognostic stratification of RCC [192, 193].

In recent years, the widespread application of immunotherapy for tumors has also brought life to a wide range of tumor patients. Along with the development of immunotherapy, immune-related research in tumors has also been widely carried out. M6A modifications have also been found to be associated with RCC-related immune features [194, 195]. These findings will help in the personalized immunotherapy of RCC patients and further prognosis assessment. Zhong, Liu [194] identified three M6A clusters and established M6A scores by a comprehensive analysis of M6A modification patterns and immune status in 513 ccRCC patients. This study confirmed the correlation between M6A modifications and immune status of ccRCC and further validated the prognostic value of the M6A score in programmed cell death protein 1 (PD-1) blockade therapy in patients with advanced ccRCC. The three subtypes of ccRCC identified by consensus clustering of M6A regulators were found to differ in terms of overall survival. Among them, one group had a poorer prognosis and higher immune activity. Further correlation analysis and validation showed that RBM15B expression in this group was negatively correlated with multiple immune characteristics [195]. In addition, M6A-associated LncRNAs have been found to be associated with the immune profile of RCC and can be taken as a prognostic marker [196, 197].

Drug-targeted therapies are more specific with less side effects than traditional therapies and have greatly improved the quality of survival of tumor patients. However, in recent years, targeted therapies have also faced problems such as drug resistance and drug sensitivity. A first-line target for RCC, sunitinib is a multi-target receptor tyrosine kinase (RTK) inhibitor. Although it is widely used, studies have shown that 10%-20% of patients with advanced disease develop significant drug resistance [198].Chen, Lu [198] screened for genes associated with sunitinib resistance by RNA sequencing and M6A sequencing. Further validation revealed that METTL14-mediated M6A modification of TRAF1 was involved in sunitinib resistance through regulation of apoptosis and anti-angiogenic effects. Similarly, M6A reader YTHDC1 is involved in ccRCC progression and regulates sunitinib sensitivity by targeting ANXA1 under the regulation of the YY1/HDAC2 complex [199]. Li et al. constructed an M6A-associated LncRNA prognostic model through a comprehensive analysis of ccRCC-related data in the TCGA database. The prognostic model can be used not only to predict the prognosis of ccRCC patients, but also to screen candidate drugs targeting the M6A-related lncRNA profile in combination with the drug sensitivity database [200].

## Conclusion

In recent years, with the development and application of high-throughput sequencing technology, the role of M6A modifications in various diseases has been widely studied in depth. Here, we systematically summarize the research on M6A modification in renal disease in recent years. Basic and mechanistic studies have identified the signaling pathways involved in M6A modifications in renal diseases and provided potential specific targets for future clinical treatments. And bioinformatics analysis not only clarifies the direction for further research, but also serves an increasingly important role in the prediction of clinical prognosis and other aspects. These studies have laid the foundation for the future search for novel and effective treatments.

## Data Availability

The authors confirm that the data support the findings of this study are available within the article.

## Abbreviations

M6A: N6-methyladenosine
mRNA: Messenger RNA
CKD: Chronic kidney disease
AKI: Acute kidney injury
MeRIP-Seq: Methyl-RNA immunoprecipitation and sequencing
ZFD: CCCH-type zinc-binding motifs
MTC: M6A methyltransferase complex
SAM: S-adenosyl-L-methionine
ELAVL1: ELAV-like RNA-binding protein 1
ADR: Adriamycin
Sirt1: NAD + -dependent deacetylase
TFA: The total flavones of *Abelmoschus manihot*
DKD: Diabetic kidney disease
SGLT2: Sodium-glucose cotransporter–2
ManNAc: Sialic acid precursor N-acetylmannosamine
FPS: Fucoidan
YAP: YES-associated protein
TGF-β: Transforming growth factor β
TAZ: Transcriptional coactivator
EMT: Epithelial-mesenchymal transition
EndoMT: Endothelial-mesenchymal transformation
SV40-MES-13: High-glucose-treated mouse mesangial cells
H3K36: Histone 3 lysine 36
LncRNA: Long chain non-coding RNA
GECs: Glomerular endothelial cells
MCs: Glomerular mesangial cells
TECs: Tubular epithelial cells
RCC: Renal cell carcinoma
ccRCC: Renal clear cell carcinoma
KIRC: Renal clear cell carcinoma
SHMT2: Serine hydroxy methyltransferase 2
miRNAs: MicroRNAs
